# Validity of a Diagnostic Scale for Acupuncture: Application of the Item Response Theory to the Five Viscera Score

**DOI:** 10.1155/2013/928089

**Published:** 2013-04-04

**Authors:** Taro Tomura, Kouichi Yoshimasu, Jin Fukumoto, Shigeki Takemura, Shunji Sakaguchi, Nobuyuki Miyai, Kazuhisa Miyashita

**Affiliations:** ^1^Department of Oriental Medicine, Kansai Vocational College of Medicine, 6-18-13 Karita, Sumiyoshi-ku, Osaka 558-0011, Japan; ^2^Department of Hygiene, School of Medicine, Wakayama Medical University, 811-1 Kimiidera, Wakayama 641-8509, Japan; ^3^Acupuncture-Moxibustion and Sports Trainer Science, Kansai University of Health Sciences, 2-11-1 Wakaba, Kumatori, Sennan, Osaka 590-0482, Japan; ^4^School of Health and Nursing Science, Wakayama Medical University, 580 Mikazura, Wakayama 641-0011, Japan

## Abstract

In acupuncture therapy, diagnosis, acupoints, and stimulation for patients with the same illness are often inconsistent among between Traditional Chinese Medicine (TCM) practitioners. This is in part due to the paucity of evidence-based diagnostic methods in TCM. To solve this problem, establishment of validated diagnostic tool is inevitable. We first applied the Item Response Theory (IRT) model to the Five Viscera Score (FVS) to test its validity by evaluating the ability of the questionnaire items to identify an individual's latent traits. Next, the health-related QOL scale (SF-36), a suitable instrument for evaluating acupuncture therapy, was administered to evaluate whether the FVS can be used to make a health-related diagnosis. All 20 items of the FVS had adequate item discrimination, and 13 items had high item discrimination power. Measurement accuracy was suited for application in a range of individuals, from healthy to symptomatic. When the FVS and SF-36 were administered to other subjects, a part of which overlap with the first subjects, we found an association between the two scales, and the same findings were obtained when symptomatic and asymptomatic subjects were compared regardless of age and sex. In conclusion, the FVS may be effective in clinical diagnosis.

## 1. Introduction

Traditional Chinese Medicine (TCM) originates from *the Huangdi's Internal Classic*, which was written in around the 2nd century BC. According to this book, the five elements (e.g., the human organs: liver, heart, spleen, lung, and kidney) are created from *yin* and *yang,* which are the foundations of all parts of the world. Qi and blood were described as flowing out from the five viscera, traveling through meridians, and connecting the acupoints on the body surface. The method of examination is also stated in the book [1, 2]. Unlike western medicine, which clearly differentiates health and illness, the concept of health in TCM denotes a state where all body components are in good balance [3]. It is considered that an imbalance is caused by the stagnation of qi and blood flow, and acupuncture is performed on the acupoints in order to resolve the stagnation.

Acupuncture was introduced to Japan around the 6th century [4]. In the following 1500 years, Japanese acupuncture has undergone a unique development in an isolated environment. Meridian Therapy (MT) is a therapy unique to Japanese acupuncture with an approach based on the theories described in ancient Chinese literature [5]. Instead of directly controlling the flow of qi and blood traveling through the meridians, MT aims to restore balance of the five viscera that control the amount of qi and blood flowing through the meridian. Hence, five viscera diagnosis is very important in Japanese acupuncture therapy.

Today, acupuncture therapy is practiced widely throughout the world, particularly in East Asia, and it is recognized as a form of complementary and alternative medicine (CAM) [6]. In acupuncture therapy, diagnosis, acupoints, and stimulation methods for a patient or patients with the same illness often vary between TCM practitioners [6–8]. Scientists are conducting intervention studies for specific diseases to evaluate the effectiveness of acupuncture therapy. However, as acupoints and stimulation methods used in the interventions vary for the same illness as mentioned earlier, the focus of these studies is usually the assessment of therapeutic effectiveness [9–11]. Their effectiveness may be affected by the variation of intervention methods. This is in part due to the paucity of evidence-based diagnostic methods in TCM. As a result, TCM cannot but has allowed the existence of various intervention methods. While a standardized diagnostic method to elucidate the effectiveness of acupuncture therapy could eliminate the advantage of TCM, developing a common diagnostic scale might on the other hand promote research and bring acupuncture therapy into the mainstream. 

We have been developing the “Five Viscera Score (FVS)” as a diagnostic scale by selecting, on the basis of statistical analysis, symptoms of the five viscera from leading TCM literature of the last 2000 years [12–14]. Like most typical scales, the FVS was created by conducting an exploratory factor analysis of the data collected from the target population and generalized based on coefficients such as Cronbach's alpha in classical test theory (CTT). Scales with higher accuracy created using CTT may not be applied to other populations, since the scale based on CTT is greatly representative of the population from which the scale was created. This dilemma is common not only in the FVS but also in all scales that are validated with CTT. In recent years, Item Response Theory (IRT) has been used so as to evaluate questionnaire items and specific properties of individuals, which enables researchers to use questionnaires without being restricted to use in the population from which the questionnaires were created. The advantages of IRT are the interchangeability of questionnaire item, the ability to develop scales as questionnaire items, and the standardization of individual traits. Accordingly, many scales are validated with IRT [15–17]. 

Scales derived from factor analysis are affected by latent variables, and IRT is a method that applies this concept and can estimate such latent ability (**θ**) hidden in individual responses and questionnaire items based on logistic functions. Hence, it is not possible to directly measure **θ** from CTT. Details of IRT have been described previously by Baker and Kim [18]. It should be noted that higher effectiveness can be expected when scales are developed, and their validity are tested with both CTT and IRT than when using CTT alone [19, 20]. There have been some studies examining diagnostic methods in TCM [21–24], but such studies to date that used IRT have been very few [25].

In our study, we first applied the IRT model to the FVS (Phase 1) to test the validity of the scale by evaluating the latent ability of the questionnaire items and individual traits. Next, the Medical Outcome Study Short-Form 36-item Health Survey (SF-36) Version 2 [26], which is a suitable instrument for evaluating acupuncture therapy, was administered to evaluate whether the FVS can be used to make a health-related diagnosis in patients (Phase 2).

## 2. Materials and Methods

### 2.1. Study Subjects and Implementation Method

#### 2.1.1. Phase 1

A total of 781 subjects (560 men and 221 women) took part in the study: 739 were students, and 42 were employees working in a vocational school in Osaka City, Japan, where the admission requirement is a high school diploma. Anonymous questionnaires were distributed to the students and employees. The study was conducted at the end of May 2010, and the collection period was 2 weeks. Additionally, the questionnaire items used in this study were based on survey sheets used to create the FVS. 

#### 2.1.2. Phase 2

This phase was conducted at the end of May 2011, using the same method as Phase 1. Two hundred and ninety-one students and 30 staff members from the same vocational school give a total of 321 subjects (208 men and 113 women). Out of these 321 subjects, 193 had also taken part in Phase 1. 

### 2.2. Ethical Considerations

The study was conducted after obtaining approval from a joint ethics committee from the Kansai Vocational College of Medicine and an external evaluation committee (H22-02, H23-08). The study subjects received a verbal and written explanation of the study objectives. Only those who expressed their willingness to participate were given a questionnaire to be completed and placed in a collection box. 

### 2.3. Questionnaire

#### 2.3.1. The Five Viscera Score

The FVS is a self-administered questionnaire consisting of 20 items related to general well-being for the previous month. It is constructed based on symptoms related to the five viscera established in TCM. To prevent biases of the questionnaire item distribution, a total of 773 symptoms related to the five viscera were selected from the TCM literature ranging from ancient to modern Chinese and Japanese texts [12]. Next, we collapsed them into 111 items excluding overlapping or unclear symptoms. Furthermore, 83 symptoms were excluded, since their standard deviations were too wide beyond their mean values (i.e., ceiling or floor effects). Then, exploratory factor analysis of 5 factors (generalized least squares method with varimax rotation) and CTT was applied for the remaining 28 symptoms, leaving 20 ones that had significant factor loadings (>0.35) [13]. Based on the five viscera function [27] and acupuncture clinicians advises, labels of “liver,” “heart,” “spleen,” “lung,” and “kidney” were assigned to each factor that has 4 subscale scores of the symptom, and the summed scores (frequency of the symptoms) were compared among the subjects. The factor loading values as well as Cronbach's *α* coefficients of subscale scores were presented in Table 2.

The questionnaire items were answered using the following 5-point Likert scale: never (0 point), rarely (1 point), sometimes (2 points), most of the time (3 points), and always (4 points). The subscale score was determined from the total score of the questionnaire items (0 to 16 points), higher scores indicating severer symptoms.

#### 2.3.2. SF-36

SF-36 Version 2 is a health-related quality of life (QOL) scale to assess subjective health status and daily life functioning [28, 29]. It is used frequently in qualitative evaluation in the field of alternative medicine. SF-36 is a self-administered questionnaire that contains 36 items related to physical and mental health status for the past month. The reliability and validity of the Japanese version have been thoroughly confirmed, and the scale has been standardized [26, 30]. SF-36 consists of the following 8 subscales: physical functioning (PF), role physical (RP), bodily pain (BP), general health (GH), vitality (VT), social functioning (SF), role emotional (RE), and mental health (MH). The scores have been standardized for the Japanese population, with the score for an average, healthy individual being 50 points. A higher score indicates a better state of health.

### 2.4. Statistical Analysis

*Phase 1: Examination of IRT Applied to the FVS*

**Figure 1:**
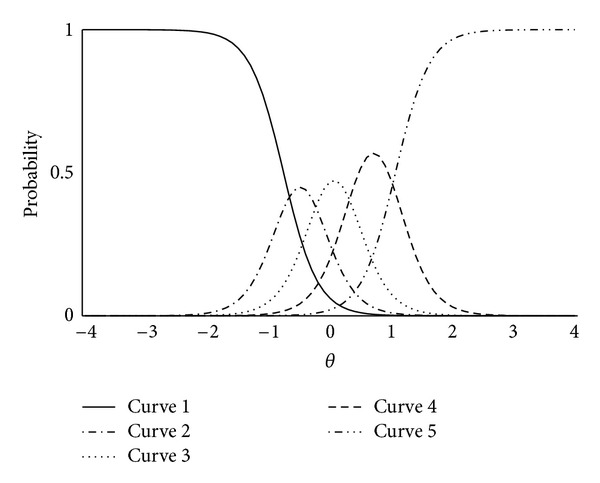
Graphic representation of Q1 of the Five Viscera Score by Item Response Theory. It shows a chart for understanding item discrimination and item difficulty of IRT. The slope of the curves represents item discrimination, and **θ** in the point of intersection of the curves represents item difficulty.

Figure 1 is a chart for understanding item discrimination and item difficulty of IRT. The five curves that were estimated by a logistic function represent each of the choices: never (curve 1), rarely (curve 2), sometimes (curve 3), most of the time (curve 4), and always (curve 5). Two fundamental parameters of IRT are item discrimination and item difficulty. The slope of the curves represents item discrimination, and **θ** in the point of intersection of the curves represents item difficulty, which means that more acute inclines suggest higher discrimination ability, and that higher **θ** values suggest more difficulty for healthy people to reply as described in the following. In an ideal question item, the graph is symmetrical with varying degrees of difficulty. 

#### 2.4.1. Item Discrimination

Item discrimination refers to the ability of an item to discriminate between individuals. The differentiation between presence and absence of symptoms is easier when scores are higher. In our study, a value of 0.35 was established as the lower limit of acceptability. A value of 1.0 or more was considered excellent. Furthermore, when estimating item discrimination, a standard error (SE) of less than 0.3 was considered excellent. 

#### 2.4.2. Item Difficulty

Item difficulty expressed the estimated degree of difficulty in answering each choice for the questionnaire item. As the FVS is answered with a 5-point scale, the degree of difficulty in representing the limitations of the choices is classified into 4 steps. The degree of difficulty also represents the intensity of the individual respondent's symptoms *(*θ*)*. Hence, both the degree of difficulty and individual latent trait is on the same axis of the **θ*,* which means that the higher the value, the more difficult it is for a normal subject to answer the question. In other words, the easier it is for a symptomatic subject to answer the question. A degree of difficulty more than −4.0 or less than 4.0 and SE less than 0.3 derived at the time of estimation was considered satisfactory.

#### 2.4.3. Test Information Curve (TIC)

The test information curve (TIC) as a scale and individual's latent nature is a graphical representation of measurement accuracy of each **θ** and is comparable to the reliability coefficient in CTT. Test information is shown on the vertical axis and **θ** on the horizontal axis, which represents the range of participants that could use the FVS. The location where **θ** is 0 represents an average subject that corresponds to a healthy individual in the FVS. 

#### 2.4.4. Relationship of Individual *θ* and Raw Subscale Score

Although the FVS is evaluated using the total item score for each subscale, test subjects with the same raw subscale score may have a different response from **θ**. The validity of the raw subscale score for the FVS was confirmed by the correlation between the raw score and the estimated **θ**. These analyses were conducted separately for gender and ages (adolescents and young adults aged teens and twenties and adults aged thirties or more), since individual **θ** and raw subscale score might be affected by those factors.

*Phase 2: Evaluation of FVS and Health-Related QOL Scale*

 Using SF-36 as an external criterion, we examined whether the FVS (i) can be used in health-related diagnosis, and (ii) can differentiate patients with and without symptoms. 

Statistical analysis for the outputs of **θ**, item discrimination, item difficulty, and TIC was performed using Kumagai's EasyEstGRM Version 0.3.6. Mann-Whitney *U* test and Spearman's rank correlation test were conducted using IBM SPSS 18. Generally accepted values [17, 20, 31] were used as a standard to determine whether each IRT item was satisfactory. For other values, statistical significance level was set at 5%.

## 3. Results

### 3.1. Phase 1

Questionnaire responses were received from 727 subjects (93.1%). A total of 133 subjects were excluded for the following reasons: missing data (89 subjects), entry error (2 subjects), and symptomatic patients who answered “always” or “most of the time” to the question “I am currently seeing a doctor for an illness” and “I am currently taking a medication” (42 subjects). Thus, a total of 594 healthy subjects (76.1%) took part in the study, 430 men (72.4%) and 164 women (27.6%). The characteristics of the subjects are shown in Table 1. The ages (mean ± SD) of men and women were 27.5 ± 8.0 and 26.0 ± 8.5 years, respectively. Regarding educational background, 36.3% of men and 38.5% of women had graduated from a two-year college or a higher academic institution. As for working hours, 77.7% of men and 51.8% of women worked 4 or more days per week. 

#### 3.1.1. Item Discrimination

As shown in Table 2, all item discrimination values reached the lower cut-off level of 0.35. Furthermore, 13 of 20 items exceeded the 1.0 level for item discrimination of which at least two or more items were included in the each subscale. 

#### 3.1.2. Item Difficulty

The average item difficulty for items greatly varied (Table 2). The questionnaire items with the highest average item difficulty value for each subscale were the question Q4 “I have migraine headaches (headaches)” for liver, Q7 “I have a lot on my mind and am not able to enjoy anything” for heart, Q12 “I don't have much energy in the morning” for spleen, Q16 “I get the hiccups” for lung, and Q19 “My memory has deteriorated” for kidney. When the subject's response was “always” for a questionnaire item that had a high average item difficulty, the symptom of the viscera related to that question was thought to be of greater severity. The item difficulty for Q4 (*b*4: 5.32) and Q16 (*b*3: 5.95, *b*4: 7.30) were particularly high. The SE for *b*4 of Q16 exceeded the standard value of 0.3. 

#### 3.1.3. Test Information Curve (TIC)

**Figure 2:**
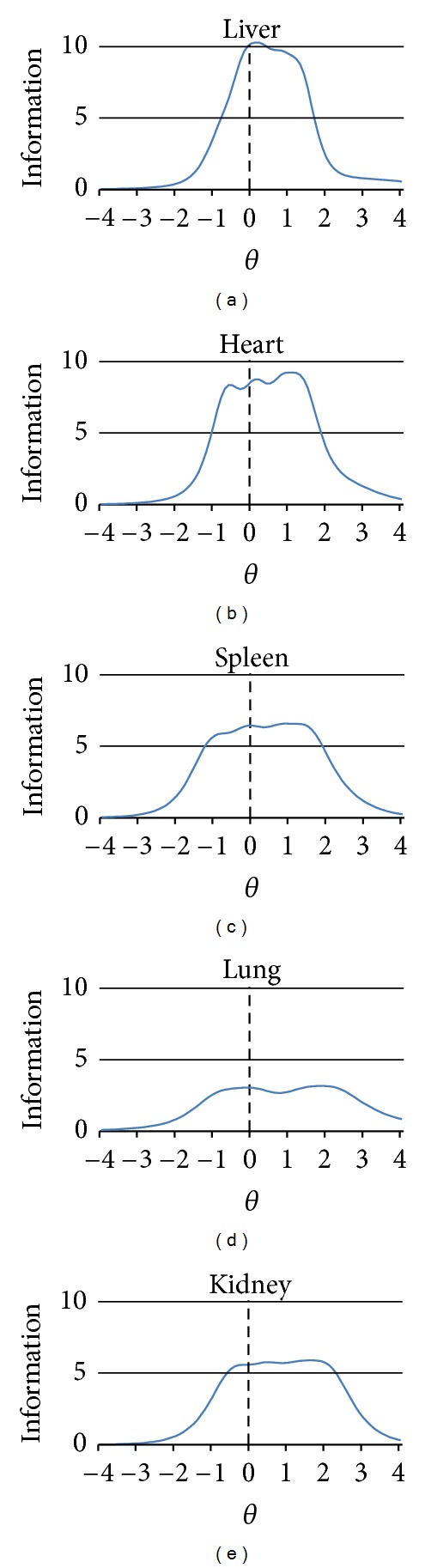
Test information curves of the Five Viscera Score. It shows the quantity of measurement information from the subscales resulting from the application of IRT. The subscales are expected to be applied to those who are in that range. All the effective ability ranges straddled zero and extended in the positive direction.

Figure 2 shows the quantity of measurement information from the subscales resulting from the application of IRT. The amount of information for adjacent **θ**s was compared, and the distance between the points where that measurement information most increased and most decreased maximally in TIC was defined as the effective ability range of the **θ**. The subscales are expected to be applied to those who are in that range. The ranges were as follows: liver (−0.50 to 1.60), heart (−1.00 to 1.80), spleen (−1.50 to 2.00), lung (−1.50 to 2.80), and kidney (−1.00 to 2.60). All the effective ability ranges straddled zero and extended in the positive direction. The greatest measurement information was as follows: 10.28 for liver, 9.24 for heart, 6.59 for spleen, 3.18 for lung, and 5.89 for kidney.

#### 3.1.4. Relationship of Individual *θ* and Raw Subscale Score (Table 3)

The correlation coefficients for **θ** and raw scores for each subscale ranged between 0.77 and 0.95 in men and between 0.92 and 0.97 in women, indicating strong associations, and the correlation was significantly stronger for women than for men in whole subjects. Fewer gender differences between individual **θ** and raw subscale score were observed in adults compared to adolescents and young adults, while women showed higher values in both generations. As with whole subjects, strong correlations between individual **θ** and raw subscale score were observed in men and women in both generations. 

### 3.2. Phase 2

A total of 302 subjects (94.1%) responded to the questionnaires. Twenty-six subjects were excluded due to missing data, and the remaining 274 subjects (85.4%) were eligible for the analysis. There were 175 male and 99 female subjects, with a mean age of 28.6 ± 7.8 years and 28.5 ± 8.6 years, respectively. Of the 274 subjects, 120 men and 66 women had participated in Phase 1. The raw subscale scores of the FVS from the Phase 1 results were used in Phase 2.

#### 3.2.1. Gender Difference in FVS and SF-36

As with Phase 1, a comparison was made to determine the presence of any gender difference in the perception of health in SF-36 compared with the FVS. Average values for women were lower than for men for all SF-36 health-related QOL subscales in whole subjects (data not shown). A significant gender difference was apparent in the following: RP (47.30 ± 11.65 in men versus 45.01 ± 12.25 in women, *P* = 0.040); BP (49.25 ± 10.04 versus 43.58 ± 10.60, *P* < 0.001); SF (48.83 ± 11.35 versus 45.55 ± 13.13, *P* = 0.031); and RE (47.95 ± 11.40 versus 43.68 ± 13.08, *P* = 0.003). Similarly, there was a marginally significant gender difference for VT (44.03 ± 10.28 in men versus 41.38 ± 11.15 in women, *P* = 0.065) and MH (44.98 ± 10.60 versus 41.95 ± 11.56, *P* = 0.054). As in Phase 1, the average FVS subscale scores for women were higher, indicating a severe symptomatic state compared with men. There was a significant gender difference for the liver (6.38 ± 3.64 for men versus 7.40 ± 3.44 for women, *P* = 0.022), and a similar trend was observed for the kidney (6.07 ± 3.28 versus 6.97 ± 3.31, *P* = 0.051), although this did not reach statistical significance. 

#### 3.2.2. Correlation between FVS and SF-36 (Table 4)

To determine whether the FVS can be used as a health-related diagnostic scale, its correlations with SF-36 scores are represented as validity coefficients in Table 4. For both men and women, all FVS subscales were significantly correlated with more than one SF-36 subscale in whole subjects. The heart, spleen, and kidney subscales of the FVS had a strong relationship with SF-36 subscales; specifically, heart and MH in men, heart and VT in women, and spleen, MH, and VT in women had correlation coefficients exceeding 0.60, indicating strong associations. Similar results were observed when dividing the subjects into two generations except that no significant correlations were observed between lung and SF-36 subscales in adult women. 

#### 3.2.3. Distinction between Symptomatic and Asymptomatic Patients on FVS (Table 5)

Lastly, for the poor healthy group (subjects who responded “always” or “most of the time” to the questionnaire items “seeing a doctor for an illness” and “taking medication for an illness”) and the healthy group (all other subjects), results for the FVS and the SF-36 were compared, as shown in Table 5. For both men and women, the poor healthy group scored high on all subscales of the FVS and low on all subscales of the SF-36. In both the FVS and SF-36, there was a clear difference between poor healthy and healthy groups for women but not in men. Similar results were observed when comparing adolescents and young adults with adults (data not shown). 

## 4. Discussion

To the best of our knowledge, this is the first study to apply IRT to FVS, a TCM diagnostic scale, evaluating the ability of the questionnaire items to identify individual latent traits. 

As mentioned previously, by removing the restriction to use caused by a population, IRT allows standardization of questionnaire items and the individual's traits that was not possible with CTT. IRT also allows the evaluation of questionnaire items on an individual basis and allows interchangeability. As there is no consistency in TCM diagnostic methods, different acupoints and methods have been used in clinical intervention studies of acupuncture to date. This has complicated the evaluation of effectiveness and hindered the collection of reliable evidence. In general, TCM uses the following four methods to formulate a comprehensive diagnosis for patients: inquiry, inspection, auscultation and olfaction, and palpation [5, 22]. Hence, it is difficult to determine whether it is possible to make a diagnosis based merely on the FVS, which is an inquiry method. However, if application of the FVS to acupuncture studies results in enhanced repeatability, it is possible that the FVS could become a standard part of the inquiry process. The FVS can be used in all fields of TCM and CAM where the state of the five viscera is evaluated in the diagnosis. Furthermore, the FVS may prove to be useful in combination with other TCM diagnostic methods [21–24] that have been under consideration. 

Excellent item discrimination (exceeding 1.0) was seen for 13 of 20 items (65.0%) of the FVS. In a previous study that applied IRT to the Beck Depression Inventory (BDI) [17], which is widely used in the diagnosis of depression, 9 out of 21 items (42.9%) demonstrated item discrimination values exceeding 1.0. We can accordingly say that the FVS has relatively high item discrimination. Using these 13 items, we can set the cut-off point for deciding the presence or absence of symptoms. Moreover, for each subscale, when a subject scores highly on an item with the highest average item difficulty, this indicates severe illness of that “viscus.” Greater item difficulties and variability exceeding the standard were observed for Q4 of liver and Q16 of lung subscales in particular, and respondents with stronger symptoms found it easier to answer these items compared with other items. The fact that questionnaire items on the FVS showed a variety of item difficulty indicates that the instrument can appropriately evaluate subjects who have various latent symptoms. 

Regarding TIC (Figure 2), which indicates measurement accuracy of the FVS, all the effective ability ranges straddled zero and extended widely in the positive direction. Hence, we demonstrated that the FVS is able to measure healthy and symptomatic individuals as well as those in a suboptimum state of health. In other words, the FVS can be used for screening healthy subjects and as a diagnostic tool for those with suboptimal health and symptomatic subjects. Furthermore, we found that the liver and lung have completely opposite properties. The liver yielded more measurement information than the lung; however, the range of application for subjects was limited. Compared to the liver, on the other hand, the lung yielded low measurement information but had a wider range of application. For instance, while there were few symptomatic subjects for the liver compared with the lung, a high score for the liver indicates with certainty the presence of a liver symptom. Similarly with the lung, while anybody with a cold becomes symptomatic, this viscus has no uniform symptoms. Hence, the TIC may be demonstrating differences in the properties of the five viscera.

If the responses significantly changed in terms of **θ**, the FVS scores had to be converted to **θ** every time the FVS was used, as **θ** cannot be observed directly in CTT. This was a possible obstacle for clinical application. However, we found a high correlation between **θ** and raw subscale scores, proving that raw subscale scores can be used in the FVS as they are, regardless of age and sex. 

In order to evaluate the external validity of the FVS, the SF-36 was administered to another subjects, a part of which overlap with the first subjects. Although there was a gender imbalance of participants with fewer women recruited, the results of both scales were consistent in showing that women have a lower subjective perception of health than men. 

Typically, when the external validity of a scale is examined, it is unnecessary to develop a new scale when the association between all items of the external standard scale and the scale that needs validation is extremely strong. Further, FVS and SF-36 show significant correlation in many items while all of which were not completely consistent. From this perspective, there is significance in developing the FVS. In addition, when we separated and compared the results of symptomatic and healthy subjects, many more differences among women could be observed in both scales, regardless of age and sex. These findings suggest that the FVS can be used for health-related diagnosis including gender differences. 

The FVS is a scale that gives objectivity to TCM diagnosis which has been used to rely on the TCM practitioner's subjective observations. TCM aims to treat those with “suboptimal health,” to prevent illness [3, 32]. In other words, prevention is considered the ultimate form of treatment in TCM. “Suboptimal health” in Western medicine is the susceptibility period prior to becoming ill. Health complaints during that period are predominantly subjective symptoms of indefinite complaint encountered in daily life [32]. Most items that compose the FVS are indefinite complaint. Since the chief complaint is the most important sign of illness both in Western medicine and in TCM, the results of our study are pertinent as we demonstrated that the symptoms related to the five viscera of TCM can be effectively used in health assessments in the Western medical field. As suggested by Schiff et al. [33], cooperation between Western medicine and CAM is important. The FVS can act as a bridge, not only for TCM practitioners, but also between eastern and Western medicine, in supplying mutually beneficial information to both sides. 

One limitation of this study was the large difference in the numbers of male and female subjects. Moreover, differences in the FVS according to age were not examined especially between young and elderly people, since the majority of the subjects were under 50. However, item discrimination as well as difficulty is not affected by individual traits such as gender or age. Further study for evaluating individual **θ** and raw subscale score as well as their correlation with SF-36 has been launched among elderly community dwellers aged 50 or more. The reliability and validity of the scale need constant examination in order to evolve and generalize the scale. In this regard, it should be noted that the limited range of application for liver and the low measurement accuracy for lung may be affecting the results of Q4 (liver) and Q16 (lung). The results of this study will be useful if the need to change these items arises in the future. 

## 5. Conclusion

In this study, we succeeded in applying IRT to the FVS to evaluate latent traits. All 20 items of the FVS had adequate item discrimination, and 13 items had high item discrimination power. Measurement accuracy was suited for application in a range of individuals, from healthy to symptomatic. There was also a strong correlation between the estimated latent traits and raw subscale scores, which demonstrated that the FVS scores could be used clinically without adjustment. When the FVS and a health-related QOL scale (SF-36) were administered to other subjects, a part of which overlap with the first subjects, we found a significant association between the two scales, and the same evaluation was obtained when symptomatic and asymptomatic subjects were compared. Thus, the FVS may be effective in clinical diagnosis. 

## Figures and Tables

**Table 1:** Characteristics of respondents, Phase 1.

Sociodemographic characteristics	Male (*N* = 430)	Female (*N* = 164)
	*n* (%)	*n* (%)
Age (y)		
18-19	55 (12.8)	39 (23.8)
20–29	240 (55.8)	82 (50.0)
30–39	102 (23.7)	28 (17.1)
40–49	24 (5.6)	12 (7.3)
50+	9 (2.1)	3 (1.8)
Mean (SD)	27.5 (8.0)	26.0 (8.5)
Education		
Junior college or lower	274 (63.7)	101 (61.6)
College or higher	141 (32.8)	57 (34.8)
Unknown	15 (3.5)	6 (3.7)
Work 4 days or more per week during last month		
No	96 (22.3)	79 (48.2)
Yes	334 (77.7)	85 (51.8)

**Table 2:** Item discrimination and difficulty of the Five Viscera Score by Item Response Theory. (*n* = 594).

Subscale	Item number	Item	*a* (SE)		Mean *b *	*b*1 (SE)		*b*2 (SE)		*b*3 (SE)		*b*4 (SE)		Factor loading	Cronbach's *α*
Liver															0.77
	Q1	I have a stiff neck	2.11 (0.11)		0.11	−0.76 (0.10)		−0.22 (0.07)		0.35 (0.08)		1.07 (0.13)		0.82	
	Q2	I have a pulled muscle in my neck	2.57 (0.14)		0.55	−0.14 (0.08)		0.25 (0.08)		0.76 (0.13)		1.35 (0.20)		0.81	
	Q3	I have a backache	0.77 (0.06)		1.40	−0.17 (0.05)		0.71 (0.06)		2.12 (0.09)		2.94 (0.14)		0.49	
	Q4	I have migraine headaches (headaches)	0.58 (0.05)		2.40	−0.26 (0.05)		1.10 (0.06)		3.46 (0.12)		5.32 (0.27)		0.41	
Heart															0.82
	Q5	I worry about many things	2.40 (0.12)		0.40	−0.66 (0.10)		0.09 (0.08)		0.82 (0.12)		1.35 (0.18)		0.83	
	Q6	I worry frequently	1.84 (0.10)		0.50	−0.63 (0.09)		0.23 (0.07)		0.96 (0.11)		1.46 (0.15)		0.76	
	Q7	I have a lot on my mind and am not able to enjoy anything	1.09 (0.07)		1.19	−0.38 (0.06)		0.76 (0.07)		1.86 (0.11)		2.54 (0.16)		0.57	
	Q8	I sigh often	0.80 (0.06)		0.89	−0.85 (0.06)		0.36 (0.06)		1.68 (0.08)		2.37 (0.11)		0.39	
Spleen															0.82
	Q9	I am fatigued and this is not alleviated by anything	1.76 (0.09)		0.25	−1.14 (0.11)		−0.09 (0.07)		0.72 (0.09)		1.50 (0.14)		0.69	
	Q10	I have to lie down due to fatigue	0.93 (0.06)		0.61	−1.24 (0.07)		0.02 (0.06)		1.37 (0.08)		2.32 (0.12)		0.60	
	Q11	My body feels heavy	1.62 (0.08)		0.33	−0.90 (0.09)		−0.11 (0.07)		0.83 (0.09)		1.52 (0.13)		0.55	
	Q12	I don't have much energy in the morning	1.12 (0.07)		0.83	−0.50 (0.06)		0.46 (0.06)		1.33 (0.09)		2.02 (0.12)		0.47	
Lung															0.64
	Q13	My stomach rumbles	1.45 (0.08)		0.70	−0.88 (0.09)		0.03 (0.06)		1.46 (0.12)		2.21 (0.18)		0.74	
	Q14	I feel hungry constantly	1.01 (0.07)		1.29	−0.43 (0.06)		0.77 (0.07)		1.96 (0.11)		2.85 (0.18)		0.58	
	Q15	I have a runny nose	0.61 (0.05)		1.30	−1.25 (0.06)		0.32 (0.05)		2.34 (0.08)		3.79 (0.15)		0.41	
	Q16	I get the hiccups	0.48 (0.05)		3.85	−0.09 (0.05)		2.24 (0.07)		5.95 (0.24)		7.30 (0.41)		0.36	
Kidney															0.79
	Q17	I am absent minded	1.70 (0.09)		0.80	−0.48 (0.08)		0.38 (0.07)		1.25 (0.12)		2.06 (0.20)		0.58	
	Q18	I am not energetic	1.45 (0.08)		0.98	−0.42 (0.07)		0.63 (0.08)		1.59 (0.13)		2.12 (0.17)		0.53	
	Q19	My memory has deteriorated	1.01 (0.07)		1.03	−0.34 (0.06)		0.61 (0.06)		1.60 (0.09)		2.25 (0.13)		0.49	
	Q20	I feel lethargic to the point where I have to lie down	0.96 (0.06)		0.81	−0.58 (0.06)		0.39 (0.06)		1.43 (0.08)		2.00 (0.11)		0.43	

FVS: Five Viscera Score, *a*: item discrimination, *b*: item difficulty (**θ**), *b*1: point of intersection with curve 1 and curve 2, *b*2: point of intersection with curve 2 and curve 3, *b*3: point of intersection with curve 3 and curve 4, *b*4: point of intersection with curve 4 and curve 5, and SE: standard error. Mean *b* is the average of 4 item difficulty values. Item discrimination represents the ability of an item to differentiate the subjects. Overall Cronbach's *α* 0.91.

**Table 3:** Gender differences and the correlation of a raw scale score and the latent trait *θ*.

		Raw score							Latent trait									
Generation	Subscale		Male			Female		*P *value^†^		Male			Female		*P *value^†^	Male* r *	Female* r *	Whole* r *
		*n*	Mean	(SD)	*n*	Mean	(SD)		*n* ^††^	Mean	(SD)	*n* ^††^	Mean	(SD)				
	Liver	430	4.45	(3.62)	164	6.67	(3.95)	<0.001	421	0.11	(0.85)	164	0.53	(1.07)	<0.001	0.77***	0.92***	0.83***
	Heart	430	5.00	(3.89)	164	5.98	(3.75)	0.002	418	0.12	(0.81)	163	0.26	(0.88)	0.038	0.79***	0.92***	0.83***
Whole subjects	Spleen	430	5.82	(3.82)	164	6.89	(3.81)	0.001	414	0.04	(0.92)	160	0.28	(0.87)	0.001	0.91***	0.94***	0.92***
	Lung	430	4.24	(2.64)	164	5.04	(2.75)	0.002	405	0.11	(0.98)	158	0.44	(0.98)	<0.001	0.87***	0.93***	0.89***
	Kidney	430	4.31	(3.59)	164	5.71	(3.68)	<0.001	356	0.17	(0.84)	153	0.41	(0.87)	0.003	0.95***	0.97***	0.96***

	Liver	295	4.27	(3.63)	121	6.21	(3.88)	<0.001	290	0.08	(0.86)	121	0.34	(1.01)	0.013	0.73***	0.91***	0.80***
	Heart	295	4.94	(3.85)	121	6.17	(3.82)	0.001	287	0.11	(0.81)	121	0.31	(0.89)	0.015	0.78***	0.91***	0.83***
Adolescents and young adults (18–29)	Spleen	295	5.83	(3.96)	121	6.93	(3.89)	0.005	284	0.02	(0.95)	119	0.25	(0.91)	0.008	0.90***	0.94***	0.91***
	Lung	295	4.40	(2.72)	121	5.25	(2.75)	0.004	279	0.15	(1.02)	118	0.52	(0.94)	<0.001	0.87***	0.93***	0.90***
	Kidney	295	4.24	(3.61)	121	5.63	(3.69)	<0.001	243	0.17	(0.85)	114	0.38	(0.87)	0.023	0.95***	0.97***	0.96***

	Liver	135	4.84	(3.57)	43	7.98	(3.88)	<0.001	131	0.17	(0.85)	43	1.06	(1.08)	<0.001	0.86***	0.97***	0.91***
	Heart	135	5.13	(3.98)	43	5.44	(3.54)	0.533	131	0.13	(0.81)	42	0.09	(0.86)	0.850	0.82***	0.97***	0.86***
Adults (30+)	Spleen	135	5.79	(3.51)	43	6.77	(3.61)	0.089	130	0.08	(0.84)	41	0.36	(0.76)	0.042	0.94***	0.96***	0.95***
	Lung	135	3.90	(2.44)	43	4.44	(2.71)	0.326	126	0.04	(0.87)	40	0.22	(1.09)	0.489	0.87***	0.92***	0.89***
	Kidney	135	4.47	(3.55)	43	5.93	(3.69)	0.017	113	0.17	(0.81)	39	0.50	(0.88)	0.039	0.96***	0.97***	0.96***

***P< 0.001, *r*: Spearman's rank correlation coefficient, ^†^Mann-Whitney *U* test, ^††^due to marginal maximum likelihood estimation, and **θ** cannot be determined for scores that are either 0 or 16.

**Table 4:** Correlation in male and female of the Five Viscera Score and SF-36.

Generation	Subscale	Whole (*r*)					Male (*r*)					Female (*r*)				
		Liver	Heart	Spleen	Lung	Kidney	Liver	Heart	Spleen	Lung	Kidney	Liver	Heart	Spleen	Lung	Kidney
	PF	−0.20***	−0.23***	−0.28***	−0.03	−0.28***	−0.20**	−0.21**	−0.30***	0.00	−0.32***	−0.18	−0.26**	−0.25*	−0.07	−0.18
	RP	−0.11	−0.37***	−0.30***	−0.06	−0.31***	−0.11	−0.39***	−0.28***	−0.11	−0.34***	−0.06	−0.34***	−0.35***	0.03	−0.24*
	BP	−0.40***	−0.35***	−0.41***	−0.21***	−0.32***	−0.38***	−0.31***	−0.42***	−0.22**	−0.30***	−0.36***	−0.38***	−0.38***	−0.19	−0.27**
Whole subjects	GH	−0.36***	−0.49***	−0.44***	−0.15*	−0.39***	−0.33***	−0.46***	−0.40***	−0.22**	−0.39***	−0.45***	−0.56***	−0.53***	−0.02	−0.39***
	VT	−0.33***	−0.57***	−0.59***	−0.07	−0.56***	−0.31***	−0.53***	−0.59***	−0.09	−0.55***	−0.34***	−0.63***	−0.61***	−0.05	−0.54***
	SF	−0.17**	−0.41***	−0.37***	−0.25***	−0.45***	−0.15	−0.36***	−0.32***	−0.26***	−0.40***	−0.18	−0.47***	−0.45***	−0.22*	−0.50***
	RE	−0.11	−0.46***	−0.36***	−0.13*	−0.42***	−0.11	−0.46***	−0.33***	−0.17*	−0.41***	−0.07	−0.46***	−0.40***	−0.06	−0.41***
	MH	−0.33***	−0.74***	−0.46***	−0.16**	−0.54***	−0.30***	−0.76***	−0.43***	−0.14	−0.53***	−0.33***	−0.70***	−0.49***	−0.20	−0.53***

	PF	−0.23**	−0.25***	−0.27***	0.04	−0.26***	−0.25**	−0.23*	−0.27**	0.10	−0.25**	−0.19	−0.31*	−0.26*	−0.08	−0.25*
	RP	−0.10	−0.31***	−0.26***	−0.01	−0.28***	−0.12	−0.29**	−0.16	−0.04	−0.24*	−0.04	−0.34**	−0.42***	0.03	−0.32*
	BP	−0.40***	−0.38***	−0.33***	−0.25***	−0.28***	−0.39***	−0.36***	−0.34***	−0.24*	−0.25**	−0.34**	−0.38**	−0.27*	−0.27*	−0.25*
Adolescents and young adults (18–29)	GH	−0.42***	−0.50***	−0.39***	−0.16*	−0.36***	−0.38***	−0.50***	−0.38***	−0.24*	−0.37***	−0.51***	−0.50***	−0.41***	−0.03	−0.37**
	VT	−0.31***	−0.54***	−0.53***	0.00	−0.53***	−0.30**	−0.49***	−0.53***	0.04	−0.51***	−0.33**	−0.61***	−0.54***	−0.07	−0.58***
	SF	−0.19*	−0.42***	−0.33***	−0.22**	−0.46***	−0.18	−0.38***	−0.30**	−0.18	−0.37***	−0.19	−0.47***	−0.38**	−0.27*	−0.59***
	RE	−0.16*	−0.43***	−0.41***	−0.08	−0.42***	−0.13	−0.40***	−0.30**	−0.06	−0.37***	−0.15	−0.46***	−0.56***	−0.08	−0.49***
	MH	−0.36***	−0.72***	−0.40***	−0.11	−0.53***	−0.32***	−0.72***	−0.36***	−0.02	−0.49***	−0.40***	−0.73***	−0.48***	−0.24	−0.60***

	PF	−0.14	−0.21*	−0.31**	−0.17	−0.33***	−0.11	−0.15	−0.34**	−0.18	−0.42***	−0.09	−0.25	−0.31	−0.19	−0.14
	RP	−0.11	−0.45***	−0.35***	−0.13	−0.37***	−0.10	−0.50***	−0.46***	−0.18	−0.53***	−0.05	−0.33	−0.24	0.00	−0.11
	BP	−0.40***	−0.30**	−0.55***	−0.15	−0.39***	−0.37**	−0.20	−0.55***	−0.17	−0.38**	−0.38*	−0.39*	−0.58***	−0.10	−0.34*
Adults (30+)	GH	−0.27**	−0.46***	−0.50***	−0.14	−0.44***	−0.25*	−0.34**	−0.41***	−0.16	−0.44***	−0.30	−0.68***	−0.72***	−0.07	−0.41*
	VT	−0.35***	−0.64***	−0.68***	−0.15	−0.59***	−0.33**	−0.61***	−0.69***	−0.21	−0.66***	−0.37*	−0.71***	−0.75***	−0.07	−0.48**
	SF	−0.12	−0.36***	−0.40***	−0.27**	−0.43***	−0.10	−0.29*	−0.33**	−0.37**	−0.48***	−0.16	−0.47**	−0.55***	−0.06	−0.34*
	RE	−0.05	−0.50***	−0.27**	−0.19	−0.42***	−0.08	−0.47***	−0.35**	−0.30*	−0.51***	0.10	−0.44**	−0.07	0.06	−0.25
	MH	−0.27**	−0.78***	−0.56***	−0.26**	−0.56***	−0.27*	−0.81***	−0.57***	−0.32*	−0.62***	−0.17	−0.70***	−0.55***	−0.11	−0.39*

*P < 0.05; **P < 0.01; ***P < 0.001 (*r*; Spearman's rank correlation coefficient); PF: physical functioning; RP: role physical; BP: bodily pain; GH: general health; VT: vitality; SF: social functioning; RE: role emotional; MH: mental health. Whole (*n* = 274; male *n* = 175; female *n* = 99), adolescents/young adults (*n* = 174; male *n* = 110; female *n* = 64), and adults (*n* = 100; male *n* = 65; female *n* = 35).

**Table 5:** Comparison by the presence of the symptom of the Five Viscera Score and SF-36.

		Whole			Male			Female		
Scale name	Subscale	Health condition			Health condition			Health condition		
		Good (*n* = 249)	Poor (*n* = 25)	* P *value^†^	Good (*n* = 162)	Poor (*n* = 13)	*P *value^†^	Good (*n* = 87)	Poor (*n* = 12)	* P *value^†^
		
	Liver	6.57 (3.56)	8.48 (3.62)	0.016	6.31 (3.62)	7.23 (3.96)	0.474	7.07 (3.40)	9.83 (2.76)	0.006
	Heart	6.73 (3.82)	8.96 (4.29)	0.018	6.56 (3.89)	7.77 (4.68)	0.472	7.06 (3.67)	10.25 (3.57)	0.010
FVS	Spleen	7.61 (3.67)	9.84 (4.18)	0.015	7.55 (3.60)	9.23 (4.68)	0.276	7.72 (3.82)	10.50 (3.66)	0.028
	Lung	5.00 (2.47)	5.88 (3.68)	0.349	5.02 (2.55)	5.62 (3.55)	0.634	4.97 (2.33)	6.17 (3.95)	0.414
	Kidney	6.24 (3.21)	7.96 (3.95)	0.040	5.96 (3.19)	7.46 (4.16)	0.268	6.76 (3.21)	8.50 (3.83)	0.111

	PF	51.99 (7.71)	49.15 (11.92)	0.480	52.08 (7.84)	49.75 (14.45)	0.781	51.83 (7.48)	48.50 (9.01)	0.189
	RP	46.68 (12.08)	44.42 (9.91)	0.131	47.49 (11.72)	44.98 (10.94)	0.296	45.17 (12.66)	43.82 (9.12)	0.368
	BP	47.83 (10.29)	40.96 (11.65)	0.005	49.71 (9.79)	43.51 (11.74)	0.061	44.32 (10.33)	38.20 (11.39)	0.081
SF-36	GH	51.05 (10.57)	39.61 (9.82)	0.000	50.93 (10.94)	40.55 (10.60)	0.001	51.26 (9.90)	38.59 (9.25)	<0.001
	VT	43.67 (10.17)	37.15 (13.52)	0.029	44.36 (10.18)	39.94 (11.07)	0.233	42.38 (10.09)	34.13 (15.69)	0.068
	SF	48.29 (11.47)	41.28 (16.11)	0.038	49.17 (10.81)	44.62 (16.72)	0.533	46.64 (12.52)	37.67 (15.30)	0.036
	RE	46.86 (11.85)	41.93 (14.68)	0.101	48.30 (11.24)	43.60 (12.95)	0.154	44.17 (12.53)	40.12 (16.75)	0.523
	MH	44.67 (10.36)	36.15 (14.41)	0.008	45.30 (10.25)	41.08 (14.29)	0.455	43.49 (10.52)	30.80 (13.06)	0.003

FVS: Five Viscera Score; mean (SD); SD: standard deviation; PF: physical functioning; RP: role physical; BP: bodily pain; GH: general health; VT: vitality; SF: social functioning; RE: role emotional; MH: mental health; ^†^Mann-Whitney *U* test. Poor: subjects who responded “always” or “most of the time” to the questionnaire items “seeing a doctor for an illness” and “taking medication for an illness”; good: the others excluding “poor.”

## References

[1] Veith I (1973). Acupuncture in traditional Chinese medicine. An historical review. *California Medicine*.

[2] Jenkins CS (1975). Acupuncture: practical considerations. *Journal of the National Medical Association*.

[3] Tsuei JJ (1978). Eastern and western approaches to medicine. *Western Journal of Medicine*.

[4] Kobayashi A, Uefuji M, Yasumo W (2010). History and progress of Japanese acupuncture. *Evidence-Based Complementary and Alternative Medicine*.

[5] Shudo D (1990). *Japanese Classical Acupuncture: Introduction to Meridian Therapy*.

[6] Linde K, Vickers A, Hondras M (2001). Systematic reviews of complementary therapies—an annotated bibliography. Part 1: acupuncture. *BMC Complementary and Alternative Medicine*.

[7] Pearl D, Schillinger E (1999). Acupuncture: its use in medicine. *Western Journal of Medicine*.

[8] Mist S, Ritenbaugh C, Aickin M (2009). Effects of questionnaire-based diagnosis and training on inter-rater reliability among practitioners of traditional chinese medicine. *Journal of Alternative and Complementary Medicine*.

[9] Sok SR, Erlen JA, Kim KB (2003). Effects of acupuncture therapy on insomnia. *Journal of Advanced Nursing*.

[10] Trinh KV, Phillips SD, Ho E, Damsma K (2004). Acupuncture for the alleviation of lateral epicondyle pain: a systematic review. *Rheumatology*.

[11] Mukaino Y, Park J, White A, Ernst E (2005). The effectiveness of acupuncture for depression—a systematic review of randomised controlled trials. *Acupuncture in Medicine*.

[12] Maeda M, Okayasu M, Shimoichi Y (2011). Preparation of the Five Viscera Score (First report): extraction and selection of symptoms based on ancient to modern texts. *The Journal of Japan College Association of Oriental Medicine*.

[13] Okayasu M, Maeda M, Shimoichi Y (2011). Preparation of the Five Viscera Score (Second report): exploratory factor analysis of the questionnaire. *The Journal of Japan College Association of Oriental Medicine*.

[14] Tomura T, Takemura S, Fukumoto J, Yoshimasu K, Miyashita K (2011). Reliability and validity of the Five Viscera Score. *Journal of the Wakayama Medical Society*.

[15] Hays RD, Morales LS, Reise SP (2000). Item response theory and health outcomes measurement in the 21st century. *Medical Care*.

[16] Bernstein IH, Rush AJ, Carmody TJ, Woo A, Trivedi MH (2006). Item response analysis of the inventory of depressive symptomatology. *Neuropsychiatric Disease and Treatment*.

[17] Castro SM, Trentini C, Riboldi J (2010). Item response theory applied to the Beck Depression Inventory. *Brazilian Journal of Epidemiology*.

[18] Baker FB, Kim SH (2004). *Item Response Theory: Parameter Estimation Techniques*.

[19] Pollard B, Dixon D, Dieppe P, Johnston M (2009). Measuring the ICF components of impairment, activity limitation and participation restriction: an item analysis using classical test theory and item response theory. *Health and Quality of Life Outcomes*.

[20] Buysse DJ, Yu L, Moul DE (2010). Development and validation of patient-reported outcome measures for sleep disturbance and sleep-related impairments. *Sleep*.

[21] Langevin HM, Badger GJ, Povolny BK (2004). Yin scores and yang scores: a new method for quantitative diagnostic evaluation in traditional Chinese medicine research. *Journal of Alternative and Complementary Medicine*.

[22] Ryu H, Lee H, Kim H, Kim J (2010). Reliability and validity of a cold-heat pattern questionnaire for traditional Chinese medicine. *Journal of Alternative and Complementary Medicine*.

[23] Huang C-M, Wei C-C, Liao Y-T, Chang H-C, Kao S-T, Li T-C (2011). Developing the effective method of spectral harmonic energy ratio to analyze the arterial pulse spectrum. *Evidence-Based Complementary and Alternative Medicine*.

[24] Lo L-C, Chen Y-F, Chen W-J, Cheng T-L, Chiang JY (2012). The study on the agreement between automatic tongue diagnosis system and traditional chinese medicine practitioners. *Evidence-Based Complementary and Alternative Medicine*.

[25] Bingwei C, Biyun X, Qiguang C (2011). Application of item response theory in syndrome of traditional Chinese medicine. *Chinese Journal of Health Statistics*.

[26] Fukuhara S, Bito S, Green J, Hsiao A, Kurokawa K (1998). Translation, adaptation, and validation of the SF-36 Health Survey for use in Japan. *Journal of Clinical Epidemiology*.

[27] Japan College Association of Oriental Medicine, Text Editorial Committee (1993). *Introduction of Oriental Medicine*.

[28] Khorsan R, York A, Coulter ID, Wurzman R, Walter JAG, Coeytaux RR (2010). Patient-based outcome assessment instruments in acupuncture research. *Journal of Alternative and Complementary Medicine*.

[29] Hunnicutt SE, Grady J, McNearney TA (2008). Complementary and alternative medicine use was associated with higher perceived physical and mental functioning in early systemic sclerosis. *Explore*.

[30] Fukuhara S, Suzukamo Y (2004). *Manual of the SF-36v2 Japanese Version*.

[31] Baker FB The Basics of Item Response Theory. http://echo.edres.org:8080/irt/baker.

[32] Wang LM, Zhao X, Wu X-L (2012). Diagnosis analysis of 4 TCM patterns in suboptimal health status: a structural equation modelling approach. *Evidence-Based Complementary and Alternative Medicine*.

[33] Schiff E, Frenkel M, Shilo M (2011). Bridging the physician and CAM practitioner communication gap: suggested framework for communication between physicians and CAM practitioners based on a cross professional survey from Israel. *Patient Education and Counseling*.

